# Lytic activity of phages against bacterial pathogens infecting diabetic foot ulcers

**DOI:** 10.1038/s41598-024-53317-4

**Published:** 2024-02-12

**Authors:** Legesse Garedew Kifelew, Morgyn S. Warner, Sandra Morales, David L. Gordon, Nicky Thomas, James G. Mitchell, Peter G. Speck

**Affiliations:** 1 College of Science and Engineering, Flinders University, Bedford Park, SA 5042 Australia; 2 St Paul’s Hospital Millennium Medical College, 1271 Addis Ababa, Ethiopia; 3 Infectious Diseases Unit, Queen Elizabeth Hospital, Woodville, SA 5011 Australia; 4 Discipline of Medicine, University of Adelaide, Adelaide, SA 5005 Australia; 5 AmpliPhi Australia Pty Ltd., Brookvale, NSW 2100 Australia; 6 Department of Microbiology and Infectious Diseases, College of Medicine and Public Health, Flinders University, Bedford Park, SA 5042 Australia; 7 Basil Hetzel Institute for Translational Health Research, Woodville South, SA 5011 Australia; 8 Centre for Pharmaceutical Innovation, University of South Australia, North Terrace, Adelaide, SA 5000 Australia; 9 Present Address: Phage Consulting, Sydney, NSW 2100 Australia

**Keywords:** Drug discovery, Microbiology, Diseases

## Abstract

Complications of diabetes, such as diabetic foot ulcers (DFUs), are common, multifactorial in origin, and costly to treat. DFUs are the cause of nearly 90% of limb amputations among persons with diabetes. In most chronic infections such as DFU, biofilms are involved. Bacteria in biofilms are 100–1000 times more resistant to antibiotics than their planktonic counterparts. Multidrug-resistant (MDR) *Staphylococcus aureus* and *Pseudomonas aeruginosa* infections in DFUs may require alternative therapeutic agents such as bacteriophages ("phages"). This study describes the lytic activity of phage cocktails AB-SA01 (3-phage cocktail) and AB-PA01 (4-phage cocktail), which target *S. aureus* and *P. aeruginosa*, respectively. The host range and lytic effect of AB-SA01 and AB-PA01 on a planktonic culture, single-species biofilm, and mixed-species biofilm were evaluated. In vitro testing showed that 88.7% of *S. aureus* and 92.7% of *P. aeruginosa* isolates were susceptible to AB-SA01 and AB-PA01, respectively, in the planktonic state. The component phages of AB-SA01 and AB-PA01 infected 66% to 94.3% of the bacterial isolates tested. Furthermore, AB-SA01 and AB-PA01 treatment significantly (p < 0.05) reduced the biofilm biomass of their hosts, regardless of the antibiotic-resistant characteristics of the isolates and the presence of a non-susceptible host. In conclusion, the strong lytic activity, broad host range, and significant biofilm biomass reduction of AB-SA01 and AB-PA01 suggest the considerable potential of phages in treating antibiotic-resistant *S. aureus* and *P. aeruginosa* infections alone or as coinfections in DFUs.

## Introduction

Diabetes is a chronic disease with a serious impact on the lives and well-being of individuals, families, and societies worldwide^1^. Complications of diabetes, such as diabetic foot ulcers (DFUs), are common, multifactorial in origin, and costly^2^. The global burden of DFUs is rising, affecting up to 26.1 million people each year and the lifetime incidence of a foot ulcer among persons with diabetes is estimated at between 19 and 34%^3^. DFUs are the precipitating cause for nearly 90% of limb amputations among persons with diabetes^3^ as most DFUs become infected^3,4^. Multidrug-resistant (MDR) *S. aureus* and *P. aeruginosa* remain the most common pathogens associated with DFU infections^5,6^. The two pathogens can be found together in a non-random distribution in which *P. aeruginosa* typically occupies deeper regions of chronic wounds while *S. aureus* may be found in the upper regions^7^. *S. aureus* and *P. aeruginosa* form in vivo biofilms that contribute to antibiotic resistance of these bacterial species^8^.

Biofilms, complex aggregates of organized microbial communities embedded in a matrix of bacterial extracellular polysaccharide substances (EPS), are involved in 60–80% of all chronic infections^9,10^. Biofilms reduce the efficacy of antibiotics because bacteria within biofilms exhibit altered metabolic properties as compared to planktonic bacteria^11^ and their formation is often considered a contributor to antibiotic treatment failure^10^. Bacteria in biofilms are 100–1000 times more resistant to antibiotics than in the planktonic phase^12,13^. The complex nature of the EPS and phenotypic heterogeneity of the constituent bacterial cells confer on biofilms significant resistance to chemical and immunological antibacterial agents^14,15^. EPS also protect bacteria as they reduce access of solutes to bacteria through a combination of ionic interaction and molecular sieving^16^.

The protective effects of biofilms on resident bacteria in conjunction with the emergence of MDR bacterial species and the absence of new antibiotics necessitates the discovery and development of new antimicrobial agents such as phages^17^. The activity of a phage can be narrow or broad within its bacterial species. However, the development of phage cocktails is one way to broaden the host range of phages^18^ as well as to delay phage resistance development^19^, regardless of the host antimicrobial susceptibility profile. Although many studies have examined activity of phages on single-species biofilms, few, including one of our previous studies, have evaluated phage activity on multispecies biofilms^20,21^. The current study examined the lytic activity of two phage cocktails AB-SA01 and AB-PA01, and their individual components, against antibiotic-resistant *S. aureus* and *P. aeruginosa* clinical isolates collected from DFUs, both in their planktonic and biofilm forms. The component phages of AB-SA01 and AB-PA01 are naturally occurring and obligately lytic belonging to the *Caudoviricetes* class^22^, intended to treat *S. aureus* and *P. aeruginosa* infection, respectively. The phage stocks used in this study were produced under current good manufacturing practice (cGMP) standards, met criteria in relation to appearance, potency, purity, identity, stability and other physical properties such as neutral pH, and were approved by regulatory agencies in the US and Australia for human use^23,24^.

## Results

### Identification and antimicrobial susceptibility profile of bacteria

A total of 91 *S. aureus* and *P. aeruginosa* clinical isolates were identified from 87 DFU patients who attended hospitals or health facilities in Adelaide, South Australia. Swab samples collected from four DFU patients yielded both *S. aureus* and *P. aeruginosa* isolates. Fifty-two *S. aureus* clinical isolates and two laboratory strains were identified using culture and MALDI TOF MS. More than 98% of the *S. aureus* isolates were susceptible to gentamicin, nitrofurantoin, fusidic acid, mupirocin, rifampicin, linezolid, tetracycline, and trimethoprim-sulfamethoxazole (TSM). The antibiotics that *S. aureus* isolates displayed the most resistance against were benzylpenicillin followed by erythromycin, oxacillin, and clindamycin with 77.8% (n = 42), 20.4% (n = 11), 18.5% (n = 10), and 18.5% (n = 10), respectively. The antibiotic susceptibility test also showed that 29.7% (n = 16) of the isolates displayed multiple antibiotic resistance profiles, including methicillin-resistant *S. aureus* (MRSA).

Thirty-nine clinical isolates and two laboratory strains of *P. aeruginosa* were identified through culture-based and MALDI TOF MS methods. All 41 *P. aeruginosa* isolates were susceptible to meropenem, amikacin, and tobramycin. More than 95% (n = 39) isolates were susceptible to norfloxacin and gentamicin. A higher proportion of isolates was found to be resistant to ticarcillin/clavulanic acid (48.8%, n = 20) and piperacillin/tazobactam (14.6%, n = 6). One clinical isolate was resistant to ticarcillin/clavulanic acid, piperacillin/tazobactam, ceftazidime, cefepime, ciprofloxacin and norfloxacin.

### Activity and host range of phages

**Figure 1:**
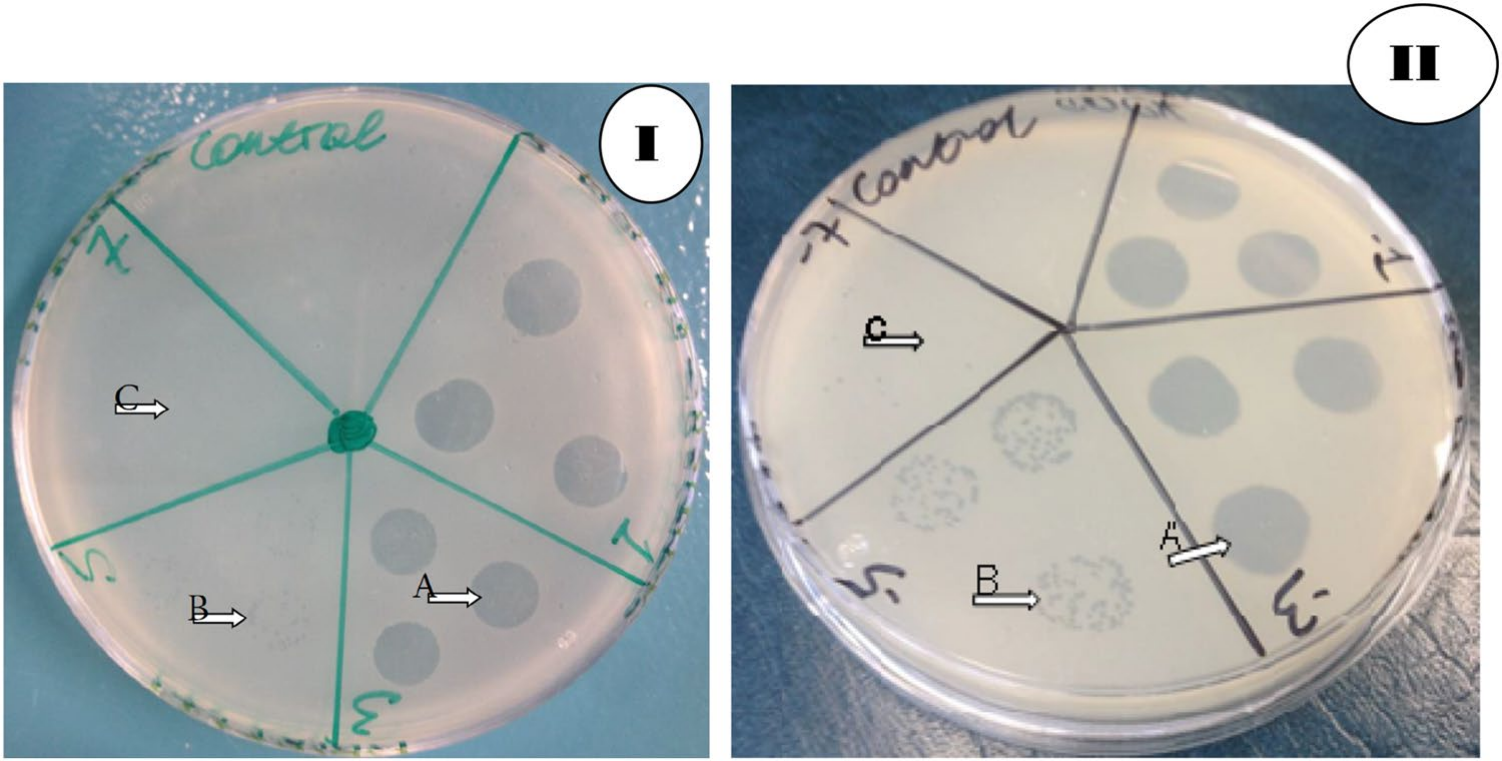
Lytic activity of AB-PA01 on *P. aeruginosa* (**I**) and AB-SA01 on *S. aureus* (**II**) isolates: A complete lysis, B intermediate lysis, and C no lysis.

**Figure 2:**
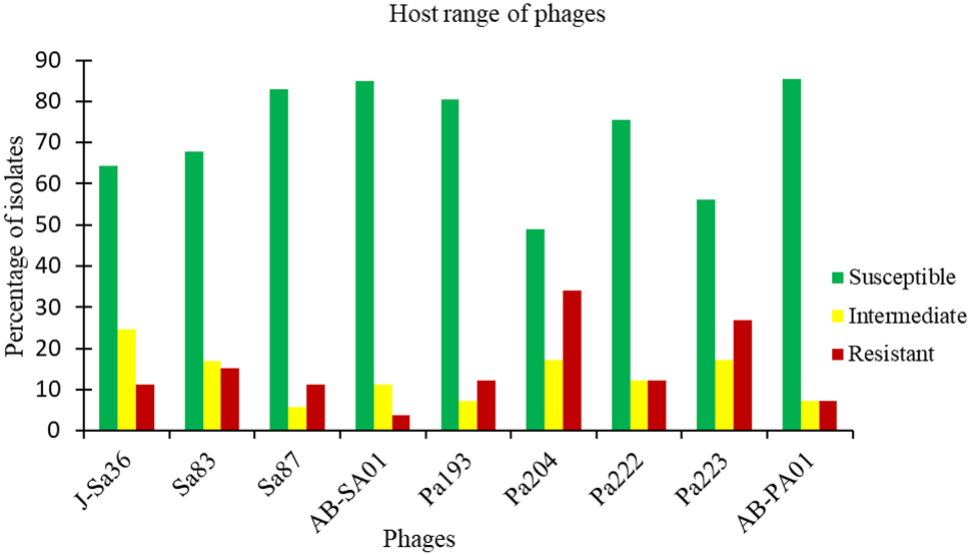
Host range of AB-SA01, AB-PA01 and their components against clinical isolates. J-Sa36, Sa83, Sa87, Pa193, and Pa204 are myoviruses, and Pa222 and Pa223 are podoviruses.

J-Sa36, Sa83, Sa87, and their cocktail AB-SA01 lysed 75.5% (n = 40/53), 83% (n = 44/53), 94.3% (n = 50/53), and 88.7% (n = 47/53), respectively, of their host isolates with full or partial lysis. Full lysis was observed in 96% (n = 45/47), 88% (n = 44/50), 85% (n = 34/40), and 82% (n = 36/44) of *S. aureus* isolates susceptible to AB-SA01, Sa87, J-Sa36, and Sa83, respectively, as shown in Fig. 1. Two *S. aureus* clinical isolates were resistant to all phages, including the AB-SA01 cocktail. All *S. aureus* isolates that were resistant to AB-SA01 were either resistant or intermediately susceptible to all component phages (Fig. 2). All MRSA isolates were susceptible to AB-SA01. There was no statistically significant difference (p > 0.05) in phage susceptibility between antibiotic-susceptible and antibiotic-resistant *S. aureus* isolates.

Of the *P. aeruginosa* isolates, 87.8% (n = 36/41), 87.8% (n = 36/41), 73.1% (n = 30/41), and 65.9% (n = 27/41) were lysed by Pa193, Pa222, Pa204, and Pa223, respectively. Five percent (n = 2/41) of isolates were resistant to all component phages of AB-PA01. AB-PA01 lysed 92.7% (n = 38/41) of the isolates, of which 89.5% (n = 34/38) lysis was complete (Fig. 1). Isolates resistant to AB-PA01 were also resistant to at least two of the component phages. The *P. aeruginosa* isolate that was resistant to multiple antibiotics was susceptible to the phage cocktail and all component phages. In most cases, isolates that showed resistance to one phage also showed resistance to at least one other phage tested. There was no statistically significant difference (p > 0.05) between myovirus (Pa193 and Pa204) and podovirus (Pa222 and Pa223) phage activity and host range, as shown in Fig. 2.

### Effect of AB-SA01 and AB-PA01 on biofilms of specific hosts

#### Treatment of single-species biofilms

**Figure 3:**
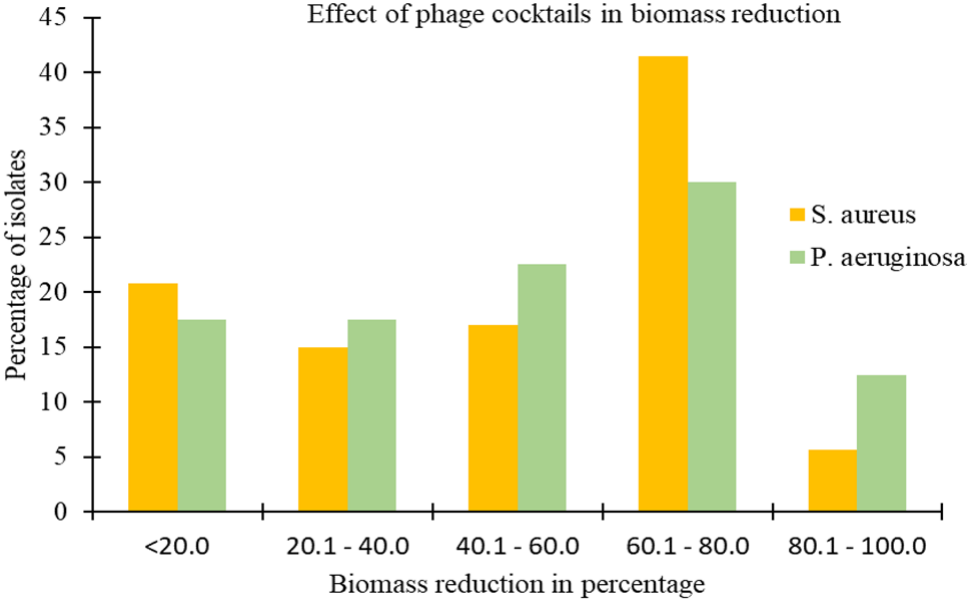
Biomass reduction effect of phage cocktails (AB-SA01 and AB-PA01) on specific host biofilms.

Of AB-SA01-treated single species biofilms, 20.7% (n = 11/53), 15.1% (n = 8/53), 17.0% (n = 9/53), 41.5% (n = 22/53), and 5.7% (n = 3/53) of the isolates’ biofilms showed insignificant, weak, moderate, strong, and very strong biomass reduction, respectively (Fig. 3). As a result of AB-PA01 treatment, 19.4% (n = 8/41), 17.1% (n = 7/41), 22.0% (n = 9/41), 29.3% (n = 12/41), and 12.2% (n = 5/41) of the *P. aeruginosa* single species biofilms showed insignificant, weak, moderate, strong, and very strong biomass reduction, respectively, as shown in Fig. 3. All of the resistant and some of the intermediately-susceptible *S. aureus* and *P. aeruginosa* isolates to their specific phage cocktail in the planktonic state showed insignificant or weak biofilm biomass reduction to the same phage cocktail treatment. In summary, 79.2% and 82.5% of biofilms of *S. aureus* and *P. aeruginosa* isolates showed significant (p < 0.05) biomass reduction compared to the PBS treatment group.

**Figure 4:**
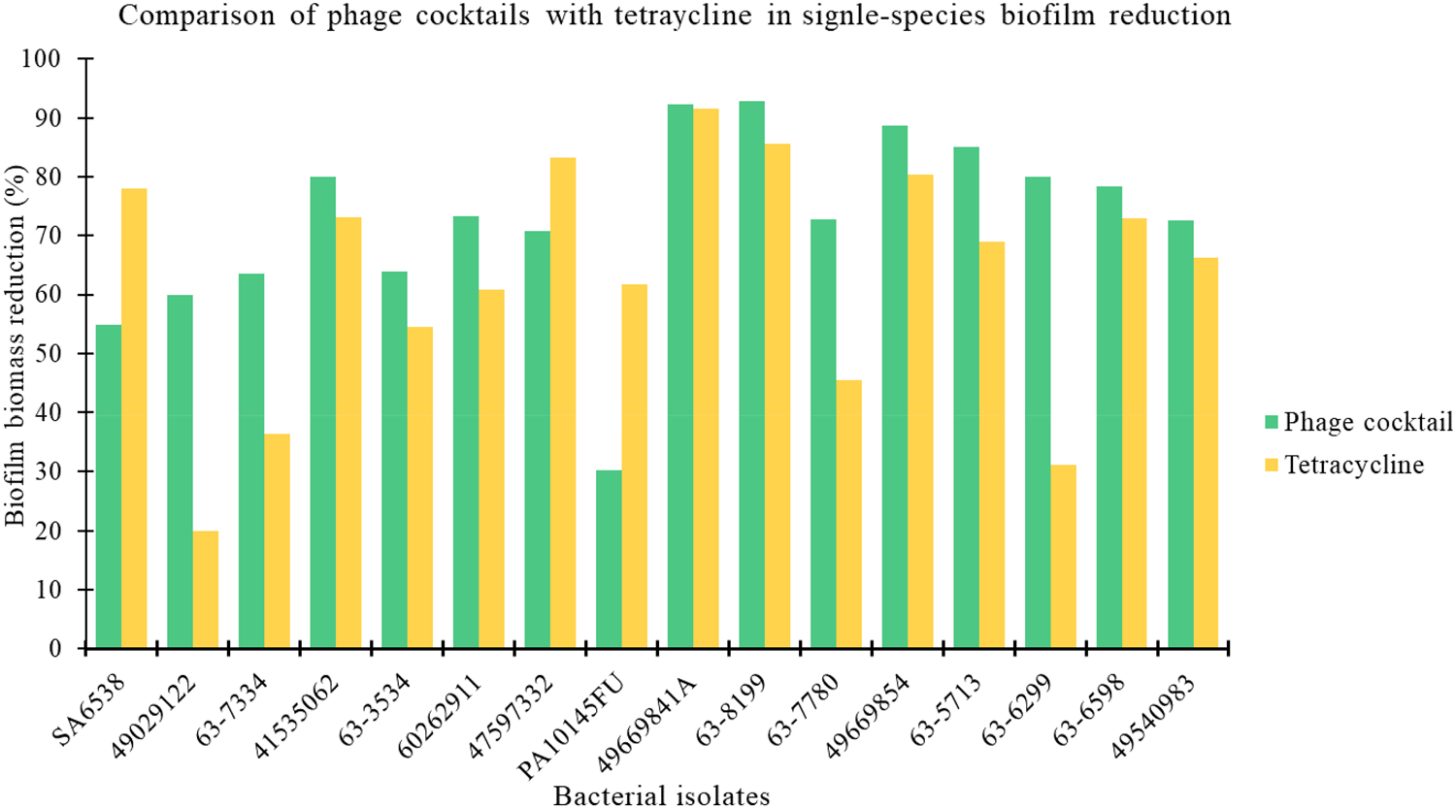
Comparison of biomass reduction effect of phage cocktails (AB-SA01, AB-PA01) with tetracycline (TTC) treatments on *S. aureus* and *P. aeruginosa* biofilm. The first seven are *S. aureus* isolates tested with AB-SA01 and the remaining are *P. aeruginosa* isolates tested with AB-PA01.

In this study, there was no significant difference (p > 0.05) between AB-SA01 or AB-PA01 and tetracycline treatment in reducing the biofilm biomass of *S. aureus* and *P. aeruginosa* isolates, as illustrated in Fig. 4. Furthermore, there was no significant difference (p > 0.05) in biofilm biomass reduction between antibiotic-resistant and antibiotic-susceptible *S. aureus* and *P. aeruginosa* isolates due to the specific phage cocktail treatment.

#### Treatment of mixed-species biofilms

Specific phage cocktail application to *S. aureus* and *P. aeruginosa* mixed-species biofilms caused a significant reduction (p < 0.05) in the host cell population compared to the PBS-treated groups (Table 1).

**Table 1:** Bacterial count (log_10_ (CFU/ml)) of *S. aureus* and *P. aeruginosa* in mixed-species biofilms after 24 h treatment.

Species used in the mixed species biofilm	No of isolates tested	Mean bacterial cell count after treatment with					Mean bacterial cell reduction			
		PBS	AB-SA01	AB-PA01	AB-SA01 + AB-PA01	Tetracycline	PBS–AB-SA01	PBS–AB-PA01	PBS–(AB-SA01 + AB-PA01)	PBS-Tetracycline
*S. aureus*	4	6.2	4.6	5.8	5.0	1.7	1.6	0.4	1.2	4.5
*P. aeruginosa*	4	6.2	6.8	4.4	4.8	1.0	0.1	2.5	2.1	5.9

Most of the tetracycline-treated cultures produced no or low viable cells of both bacterial species, showing that tetracycline was more effective than the phage cocktails or their combination in treating mixed-species biofilms. The mean bacterial cell population of each species remained unaffected when treated with the other species’ phage cocktail alone. Treatment of mixed-species biofilms using the mixture of the two phage cocktails, AB-SA01 + AB-PA01, produced similar cell density reduction as each phage cocktail alone treatment on both *S. aureus* and *P. aeruginosa* isolates.

## Discussion

*S. aureus* and *P. aeruginosa* are leading causative microorganisms of DFU infection among Gram-positive and Gram-negative bacteria, respectively^25^. In the current study, 52 *S. aureus* and 39 *P. aeruginosa* clinical isolates were identified from DFU patients. A significant proportion (16.7%) of *S. aureus* isolates were identified as MRSA in agreement with recent reports in Australia^25^ and elsewhere^26^. However, our figures are lower in comparison with the 50–60% figures from other studies, reflecting known regional variations in MRSA prevalence^27,28^. The chronic and polymicrobial nature of the DFU with frequent antibiotic exposure may partly explain the overall high rates of MRSA infection^28,29^.

Intrinsic antimicrobial resistance mechanisms encoded in the *P. aeruginosa* genome lead to high MICs for many antimicrobials^30^. About half of *P. aeruginosa* isolates tested were resistant to the anti-pseudomonal agents ticarcillin/clavulanic acid and 10–15% of the isolates were resistant to ciprofloxacin, cefepime, ceftazidime, and piperacillin/tazobactam. The antibiotic-resistance pattern of *P. aeruginosa* isolates in this study is consistent with published findings^31^.

Therapeutic phages could be alternatives to treat antibiotic-resistant infections as they kill bacterial hosts irrespective of antibiotic susceptibility^32^. We showed that at least 75% of *S. aureus* isolates were susceptible to each component phage (myoviruses) and the phage cocktail AB-SA01. Similar activity and host ranges were reported for Sa83, and Sa87 phages on *S. aureus* isolates collected from chronic rhinosinusitis patients^33^.

In this study, both the myovirus (Pa193, Pa204) and podovirus (Pa222, Pa223) phages exhibited lytic activity against a broad range of *P. aeruginosa* clinical isolates, expanding on the in vitro data set that supports their selection as candidates for clinical applications. Phage cocktail AB-PA01 demonstrated a superior host range compared with its components. The individual phages and phage cocktails used in this study were active against the high bacterial inoculum (8 log_10_ (CFU/ml)) used to mimic heavily infected wounds^34^. AB-SA01 and AB-PA01 and their components produced their lytic effect at the theoretical multiplicity of infection (MOI) of 1 which is lower than the ‘multiplicity of 10 rule’, which states that if the goal is to significantly reduce a bacterial density, it is recommended to use a phage-to-bacteria ratio of 10:1^35^.

The broad host range and strong lytic activity observed here due to phage cocktails are consistent with published reports^34,36^. There is a small possibility that the coinfection of component phages negatively affects the outcome of lytic activity when the second phage adsorption extends the latent period of the first phage^37,38^. This phenomenon could be one of the reasons for the higher host range observed in one of the phage components than the phage cocktail used in this study. The development of a phage mixture is generally preferred over a single phage as it results in a decreased rate of resistance development^39^.

A limited number of studies have shown the potential of phages to reduce or eliminate biofilms^21,40,41^. Here, AB-SA01 and AB-PA01 produced a significant decrease in biofilm biomass of isolates tested. All bacterial isolates that were resistant or intermediately susceptible to phage cocktails in planktonic assays showed little (< 10%) to no biofilm biomass reduction. AB-SA01 and AB-PA01 were more active in the planktonic state than in biofilms, as bacteria in the biofilm mode of growth are protected and more resistant to external effects than in the planktonic state^42^. The biofilm reduction capability of the two phage cocktails used in this study was not related to the antibiotic resistance characteristics of the isolates. The present study suggests phage cocktails could be alternatives or adjuncts to antibiotic treatments for *S. aureus* and *P. aeruginosa* biofilm related DFU infections.

Difficulties in growing different bacterial species together in vitro make the study of co-infections challenging, and this is true in the case of *S. aureus* and *P. aeruginosa*. Some studies indicated that co-culturing these two species negatively affects growth of both species^43,44^, but others showed that they have commensal or synergistic interactions^8,45^. Either of these phenomena might have affected the current study and could be considered the limitation of the study.

Mixed-species biofilms are complex microbial and extracellular matrix combinations that make phage therapy difficult^46^. In this study, treatment with AB-SA01 and AB-PA01 phage cocktails significantly decreased density of both *S. aureus* and *P. aeruginosa* demonstrating the phage cocktails could effectively reach and kill their hosts, consistent with our previous study^21^. This finding agrees with findings obtained when *E. co*li-specific phages were added to an *E. coli-S.* Typhimurium mixed culture^47^. The findings of this study demonstrate that phage cocktails AB-SA01, AB-PA01, and a mixture of AB-SA01 and AB-PA01 lysed their hosts in the presence of biofilms of non-susceptible species. Our results differ from an observation^48^ showing that presence of non-susceptible bacteria in mixed-species biofilms protected susceptible bacteria from phages.

In Conclusion, each phage and phage cocktail showed a broad host range of infectivity. Further, both phage cocktails demonstrated activity in single-species biofilm biomass reduction superior or similar to tetracycline, suggesting AB-SA01 and AB-PA01 are good candidates for further development to treat chronic wound infections caused by *S. aureus* and *P. aeruginosa*. There was no significant difference in phage sensitivity between antibiotic-resistant and antibiotic-sensitive clinical isolates. The lytic activity of phages, including on multidrug-resistant isolates and in mixed-species biofilms, shows AB-SA01 and AB-PA01 and their components may be a practical alternative or adjunct treatment to antibiotics in DFU infections.

## Materials and methods

### Bacterial isolates

In this experiment no human subjects or human tissues were involved. *S. aureus* and *P. aeruginosa* isolates were collected from foot ulcers on DFU patients seen in hospitals in Adelaide, South Australia. Samples were cultured on Muller Hinton agar and a pure colony of each isolate was plated on selective agar for *S. aureus* (mannitol salt agar, MSA) and *P. aeruginosa* (vancomycin-supplemented MacConkey agar) (Thermo Fisher, South Australia) as described^49^. A colony showing typical characteristics of each species on 18 h selective agar plate growth was applied to a matrix-assisted laser desorption ionization-time of flight mass spectrometry (MALDI-TOF MS) sample plate^50^. A thin film was formed by spreading the bacterial cells evenly on the plate, 1 µl 70% formic acid was applied, and left to dry at room temperature. Samples were then overlaid with 1 µl of the alpha-cyano-4-hydroxycinnamic acid (HCCA) matrix solution and allowed to dry before loading samples into the Bruker Daltonik MALDI-TOF MS Biotyper (BRUKER Pty. Ltd., Victoria, Australia) for identification^51,52^. The results were analysed using BRUKER Biotyper 3.0 software. Isolates were considered *S. aureus* or *P. aeruginosa* when the first- and second-best match organism scores were ≥ 2.00. Reference strains *S. aureus* subsp. *aureus* Rosenbach (ATCC 6538), *S. aureus* subsp. *aureus* RN4220, *P. aeruginosa* PAO1, and *P. aeruginosa* (ATCC 15692) were used for quality control.

### Antibiotic susceptibility of bacterial isolates

Following the established protocol^53^, *S. aureus* and *P. aeruginosa* isolate colonies from 24 h culture plates were diluted in 0.45% sterile phosphate buffer saline (PBS, pH = 7.0) solution and standardized between 0.5 and 0.63 McFarland turbidity standards. Following the manufacturer’s guidelines (BioMérieux, Inc., North Carolina, USA), a 280 µl of *S. aureus* or 145 µl of *P. aeruginosa* suspension was transferred into a tube with 3 ml of a 0.45% PBS solution, and the tube loaded with antimicrobial susceptibility test cards was placed in a test cassette, which was incubated in a VITEK® 2 machine (bioMérieux Australia Pty Ltd, New South Wales, Australia) for analysis (Weber et al., 2017). Antibiotics included in the susceptibility test of *S. aureus* (VITEK® 2 AST-P656) were benzylpenicillin, oxacillin, gentamicin, ciprofloxacin, erythromycin, clindamycin, linezolid, daptomycin, teicoplanin, vancomycin, tetracycline, nitrofurantoin, fusidic acid, mupirocin, rifampicin, and trimethoprim-sulfamethoxazole (TSM). The susceptibility test for *P. aeruginosa* (VITEK® 2 AST-N246) contained ticarcillin/clavulanic acid, piperacillin/tazobactam, ceftazidime, cefepime, meropenem, amikacin, gentamicin, tobramycin, ciprofloxacin, and norfloxacin. The VITEK® 2 system software 8.01 was used for analysis.

### Phages

Both phage cocktails and their components were provided by AmpliPhi Australia Pty Ltd, Brookvale, NSW, Australia (now Armata Pharmaceuticals, Inc.). *S. aureus* phage cocktail AB-SA01 is composed of three naturally occurring and obligately lytic myoviruses, closely related to *Staphylococcus* phage K, designated J-Sa36, Sa83, and Sa87^22,23,54^, and which we have used to treat *S. aureus*-infected lesions in a mouse model^55^. *P. aeruginosa* phage cocktail AB-PA01 is composed of four phages designated Pa193 and Pa204 (myoviruses), and Pa222 and Pa223 (podoviruses). All these phages belong to the *Caudoviricetes* class^22,54,56^. All phages were determined to be strictly lytic, sequencing confirmed that phages were free of genes encoding bacterial virulence factors or drug resistance^23,56^. Prior to each assay, the stock suspension of each phage was titrated against selected host bacterial strains using the soft agar overlay assay, as described below. The mean phage titre ranged from 9.0 to 9.3 log_10_ (total plaque forming units (PFU)/ml) for AB-SA01 and its constituents and from 9.2 to 9.5 log_10_ (PFU/ml) for AB-PA01 and its constituents.

### Phage efficacy and host range test

To evaluate in vitro activity and host range of the phages, spot tests were performed as described (Alves et al.^41^). Briefly, 100 µl of an overnight culture of each bacterial isolate was mixed with 3 ml of tryptic soy soft agar (TSA, 0.6% agar) at 42 °C, poured onto pre-warmed (37 °C) TSA (1.2% agar) plates and evenly distributed. Ten microliters of each phage solution, including individual phages and phage cocktails, was serially diluted with tryptic soy broth (TSB). Ten microliters of 10^–1^, 10^–3^, 10^–5^, and 10^–7^ dilutions were spotted onto bacterial lawns in triplicate. The results were considered valid when the triplicates yielded similar results, and the mean was taken as final. Sterile PBS was used as a negative control and plates were left in a biosafety cabinet II for 30 min to dry. Plates were incubated inverted at 37 °C aerobically overnight and examined the following day. Susceptibilities were categorized based on AmpliPhi Australia Pty Ltd guidelines as follows: (i) resistant if weak or no activity was seen, (ii) intermediate when faint and turbid spots were observed, and (iii) susceptible when a clear spot with no bacterial growth within the spot was observed.

### Measurement of the activity of phage cocktails on in vitro single-species biofilms

Biofilms of *S. aureus* and *P. aeruginosa* isolates were developed using an established model and measured by optical density at 600 nm^57^. *S. aureus* and *P. aeruginosa* isolates were cultured overnight on MSA and vancomycin-supplemented MacConkey at 37 °C, respectively. A single colony of each isolate was transferred into a sterile glass tube of 0.45% PBS and adjusted to 1.0 McFarland turbidity standard. The suspension was diluted at 1:100 into 1% glucose-TSB, and 150 µl of the suspension was transferred to a sterile flat-bottom 96-well Greiner CELLSTAR® polystyrene tissue culture plate (Sigma-Aldrich, New South Wales, Australia), and incubated for 48 h on a gyratory mixer of 70 revolutions per minute (rpm) at 37 °C. Plates were washed three times with sterile deionized water through gentle pipetting and air-dried. Based on biofilm-producing capacity^57^, isolates were assigned: (i) optical density (OD)600 < 0.1 non-, (ii) 0.1 ≤ OD600 < 1 weak-, and (iii) OD600 ≥ 1 strong-biofilm producers. Treatment with 180 µl of each phage cocktail (1.7 log_10_ PFU), or tetracycline (16 µg/ml for *S. aureus* and 128 µg/ml for *P. aeruginosa*, as positive control), or sterile PBS solutions (negative control) in TSB broth was applied. The minimum inhibitory concentration (MIC) of tetracycline was 128 µg/ml and ≤ 8 µg/ml for *P. aeruginosa* and *S. aureus*, respectively, as determined by VITEK® 2 test and agar and broth dilution methods^58^. These results particularly the efficacy of tetracycline on *P. aeruginosa*, were supported by studies that showed tetracycline is effective on *P. aeruginosa* at higher concentrations^59–61^. The MIC of tetracycline against *S. aureus* determined through VITEK 2 was higher than Clinical and Laboratory Standards Institute (CLSI) and European Committee on Antimicrobial Susceptibility Testing (EUCAST) standards^62,63^ and we used the higher concentration for a strong control group. The treatments were: (i) 18 µl phage mixed with 162 µl broth, (ii) 3 or 23 µg tetracycline in 180 µl broth, and (iii) 18 µl PBS in 162 µl broth. After 12 h of incubation under static conditions, the fluid portion of the culture was decanted, and the biofilm was washed twice through gentle immersion in distilled water. Biofilms were fixed using 95% methanol for 30 min, washed once with distilled water and air-dried, and stained with 190 µl of 0.2% crystal violet (CV, dissolved in deionised water) for 60 min. The excess stain was removed by gentle washing twice with distilled water, left to dry in a dark room overnight, and eluted in 200 µl of 30% acetic acid for 30 min. The eluted suspension was transferred into a new microplate using 30% acetic acid as a negative control, and OD600 was measured using a FLUOstar® Omega microplate reader (BMG LABTECH Pty. Ltd. Victoria, Australia). This was conducted on 53 *S. aureus* and 41 *P. aeruginosa* clinical isolates. Seven *S. aureus* and nine *P. aeruginosa* isolates which were strong biofilm producers and susceptible to their phage cocktails, in planktonic states, were randomly selected to compare the effect of phage cocktails with tetracycline in single-species biofilm treatment. The percentage of biofilm biomass reduction (%BK) was calculated from the absorbance of background-corrected untreated controls (IC, PBS treated) and treatments (IT) as described previously^64^: %BK = (IC–IT)/IC × 100%. The biofilm biomass reduction was categorized as: (i) insignificant; < 20%, (ii) weak; 20.1–40.0%, (iii) moderate; 40.1–60.0%, (iv) strong; 60.1–80.0%, and (v) very strong > 80% reduction.

### Phage treatment of in vitro mixed-species biofilm

The mixed-species biofilm treatments were conducted as described^34,65^ with a few modifications. Four *P. aeruginosa* and *S. aureus* isolates which were strong biofilm producers and susceptible for their phage cocktails, in planktonic and biofilm states, were randomly selected for the mixed-species biofilm treatment experiment. Briefly, 2–3 colonies of 18 h culture from MSA or vancomycin-supplemented MacConkey agar of each isolate were suspended in sterile PBS and adjusted to 1.0 McFarland turbidity standard. These suspensions were pooled at a 1:3 v/v ratio of *P. aeruginosa-*to-*S. aureus* (based on our pilot study to attain equivalent colony forming units (CFUs) from the mixed-species biofilm), and 100 µl of the pooled suspension was transferred to 10 mL 5% bovine serum albumin (BSA) nutrient broth to facilitate *S. aureus/P. aeruginosa* coexistence^66^. The final suspension was supplemented with 1% sterile glucose to facilitate biofilm development. Two hundred microlitres of the suspension were transferred into a tissue culture plate in triplicate and incubated for 48 h at 37 °C with 70 rpm agitation.

After 48 h incubation, liquid culture was removed, and plates were washed gently twice using sterile deionized water. Then, 225 μl of each treatment was applied to the respective treatment group. The treatment categories of mixed-species biofilms were: (i) AB-SA01, (ii) AB-PA01, (iii) a mixture of the two phage cocktails, AB-SA01 + AB-PA01, (iv) tetracycline (128 µg/mL, positive control), and (v) PBS (negative control). Treated biofilms were incubated statically for 12 h at 37 °C. The biofilm was washed twice using 250 μl sterile PBS through careful pipetting. The biofilm-associated cells were collected with 225 μl nutrient broth through pipetting after scraping the wall and bottom of the wells with a loop for colony count as described^46,67^. After homogenization by gentle vortexing, the cell suspension was serially diluted, 10^–1^–10^–8^, in filter-sterilized 10 mM ferrous ammonium sulphate (FAS) supplemented nutrient broth to inactivate free phages^68^. After 15 min incubation of the suspension at room temperature, serial dilution and plating were carried out. Colony count was performed after 24 h incubation at 37 °C. Plates with an estimated 30–300 colonies were selected for colony count. The mean CFU of the three plates of the same dilution was taken as final. The number of bacterial cells was calculated using the formula B = N/d where B = number of bacteria; N = average number of colonies; and d = dilution factor (Mendes et al.^49^). STATA (StataCorp, College Station, Texas) version 16 was used to evaluate the significance of each treatment and compare the treatment effect among each group. Comparison of treatment effect was also done using a one-way analysis of variance (for three or more independent groups) and paired ‘*t* test’ (for two independent groups) to confirm the STATA analysis result. A p < 0.05 value was considered statistically significant. All results were reported as the mean ± standard error of the mean and were expressed as logarithm-transformed values log_10_ (CFU/mL) over time.

## Data Availability

The datasets used and/or analysed during the current study are available from the corresponding author on request.
